# Comprehensive proteome profiling of molecular endotypes in Japanese adults with moderate-to-severe atopic dermatitis

**DOI:** 10.3389/fmed.2025.1649918

**Published:** 2025-11-20

**Authors:** Victoria Serelli-Lee, Akichika Ozeki, Christoph Preuss, Robert J. Benschop, Hitoe Torisu-Itakura, Takashi Matsuo, Jonathan T. Sims

**Affiliations:** 1 Eli Lilly Japan K.K., Kobe, Japan; 2 Eli Lilly and Company, Indianapolis, IN, United States

**Keywords:** atopic dermatitis, biomarkers, endotype, inflammation, proteomics, computational

## Abstract

**Introduction:**

Atopic dermatitis (AD) is an inflammatory skin disease that is heterogeneous in clinical presentation and biological mechanisms. Several studies have suggested biomarker-defined molecular endotypes in AD. This study aimed to characterize potential endotypes in Japanese patients with moderate-to-severe AD and comprehensively evaluate their circulating protein profiles to better understand disease etiology.

**Methods:**

Serum samples from Japanese patients with moderate-to-severe AD (*n* = 73) enrolled in a phase 3 study of baricitinib (BREEZE-AD2; NCT03334422) and samples from healthy controls (*n* = 15) were analyzed using the Olink Explore 1536 assay. Patient clusters were identified through *k*-means clustering. Differential expression analysis and weighted gene co-expression network analysis were performed for in-depth examination of proteomic profiles.

**Results:**

Two patient clusters, characterized by high (AD_HI) and low (AD_LO) inflammatory profiles, were found to be stable and reproducible. Canonical AD inflammatory mediators—including interleukin (IL)-13, IL-19, pulmonary and activation-regulated chemokine (PARC), thymus and activation-regulated chemokine (TARC), chemokine (C-C motif) ligand (CCL)22, CCL26, and CCL27—were upregulated in both clusters, with greater upregulation in the AD_HI cluster. Additionally, proteins not typically associated with AD-related inflammation were upregulated in AD_HI patients. The AD_HI cluster was associated with protein networks representing a range of immune and non-immune pathways. Dysregulated protein signatures associated with the AD_HI cluster were also correlated with skin-based disease severity scores.

**Conclusion:**

This study characterizes the circulating proteome and clinical characteristics across putative molecular endotypes in AD. The findings corroborate current knowledge on AD pathophysiology and suggest other axes of dysregulation in a subset of patients with AD. These results may support the development of personalized therapeutic approaches.

## 1 Introduction

Atopic dermatitis (AD) is a common inflammatory skin disease worldwide, characterized by heterogeneity in clinical presentation and underlying biological mechanisms (1, 2). Despite this, patients are often managed using a generalized treatment approach (3). Although significant progress has been made in understanding AD immunopathology, further insight into the molecular mechanisms underlying its varied phenotypes is an important step toward personalized medicine.

AD is mediated by T-helper (Th) cell responses (4). The interleukin (IL)-13/Th2 axis is a key pathway in AD pathogenesis, although Th1 and Th17 pathways have also been implicated (1, 2, 5). In Japan, approved targeted therapeutics for AD include monoclonal antibodies targeting the IL-4/IL-13 pathway, broad-acting small-molecule Janus kinase (JAK) inhibitors, and IL-31-targeting therapy, which is approved for the treatment of AD-associated itching (6, 7). Several thymic stromal lymphopoietin (TSLP)-targeting therapies are in advanced stages of clinical development. While these treatments can temporarily alleviate AD symptoms, their efficacy and long-term safety remain limited (8).

AD can be classified into endotypes based on the immunopathologic disease mechanism. Although still limited, a growing number of studies have characterized biomarker-defined AD endotypes using blood or skin samples and have identified regional and ethnic differences in disease mechanisms and phenotypes (1, 9–14). Upregulation of the Th2/Th17 axes has been observed in Asian patients with AD, potentially indicating a blended molecular phenotype of disease axes previously considered mutually exclusive to AD and psoriasis, respectively (9, 15). A recent analysis of skin and peripheral blood mononuclear cell samples from Japanese patients with AD demonstrated phenotype–endotype associations and variability in immune cell profiles within individuals over time (10). Factors correlating with disease severity in these patients included the serum thymus and activation-regulated chemokine (TARC), lactate dehydrogenase, and eosinophil counts—all established AD biomarkers (10). In this same study, network analysis also identified two novel transcriptome modules associated with disease progression (10).

We have previously published data describing endotypes characterized by their level of inflammation, as measured by circulating protein biomarkers (11). While there is no current consensus on which of the identified biomarker-defined endotypes is most biologically or clinically significant, these findings indicate that a systemic inflammatory state accompanies the physical inflammation observed in adult patients with AD (11). The degree of inflammation was found to correlate with disease severity to varying extents among different ethnic groups (11). Building on these data, we aimed to perform a similar study focusing on a single ethnic population.

Using computational methods, including clustering and network analysis, this study aimed to identify disease endotypes in Japanese patients with moderate-to-severe AD. Additionally, we sought to characterize clinical features associated with each identified endotype.

## 2 Methods

### 2.1 Participants and clinical data

This study included 73 Japanese patients with moderate-to-severe AD enrolled in the global phase 3 study of baricitinib (BREEZE-AD2; ClinicalTrials.gov: NCT03334422). Included patients were aged ≥18 years; had an AD diagnosis according to the American Academy of Dermatology criteria for at least 12 months prior to screening; and had moderate-to-severe AD indicated by a baseline Eczema Area and Severity Index (EASI) score ≥16, an Investigator’s Global Assessment for AD score ≥3, and body surface area involvement ≥10%. Patients also had a documented history of inadequate response or intolerance to existing topical medications. Key exclusion criteria included concomitant skin conditions such as psoriasis, eczema herpeticum, or skin infections requiring treatment with systemic or topical antibiotics or corticosteroids. Full details of the inclusion and exclusion criteria can be found on ClinicalTrials.gov.

Patient demographics and baseline clinical trial data—including age, sex, body mass index (BMI), AD clinical scores, prior therapy use, and routine safety clinical laboratory test results obtained at the same visit as the serum samples—were used in this analysis. These data are collectively referred to as clinical and laboratory data in subsequent sections. A total of 15 age- and sex-matched healthy control (HC) subjects with no allergic conditions were recruited separately, and serum samples were collected using the same procedures as for the patient cohort. The inclusion criteria for HC subjects are outlined in the Supplementary methods.

The study was conducted in accordance with the Declaration of Helsinki and Good Clinical Practice guidelines. All participants provided written informed consent before any study-related procedures and consented to biomarker testing.

### 2.2 Serum proteomic assay and data

Patients underwent a 4-week systemic and 2-week topical AD therapy washout period before clinical trial enrollment. To ensure an accurate reflection of basal biological profiles, serum samples analyzed in this study were collected after the washout period and before administration of the first trial drug, in accordance with the clinical trial protocol. Blood was collected in tubes containing a clotting agent (Becton, Dickinson and Company, Serum Separator Tube 5 mL). The tube was allowed to stand for 30 min before being centrifuged at 1,500 g for 15 min. The supernatant was obtained and stored at −70 °C until the assay was performed.

Circulating proteins were assayed using the Olink Explore 1536 panel (Olink^®^ Proteomics, Sweden, catalog #97000), a proteomics platform that combines an antibody-based immunoassay with a proximity oligonucleotide extension assay and signal detection with next-generation sequencing on the NovaSeq6000 (Illumina Inc., San Diego, United States). The panel includes four subpanels, as defined by the manufacturer: inflammation, oncology, neurology, and cardiometabolic. Sample preparation and assay procedures were performed according to the manufacturer’s specifications. The measured protein expression levels in each sample were adjusted relative to a plate control sample to yield a normalized protein expression (NPX) value. Details on protein expression normalization, data quality control (QC) procedures, and criteria are provided in the Supplementary methods. Following QC filtering, which included the selection of markers with a CoV >20%, 1,248 protein analytes were available for all downstream analysis. Olink data are referred to as proteomic or biomarker data in subsequent sections.

### 2.3 Cluster stability and reproducibility analysis

**Figure 1:**
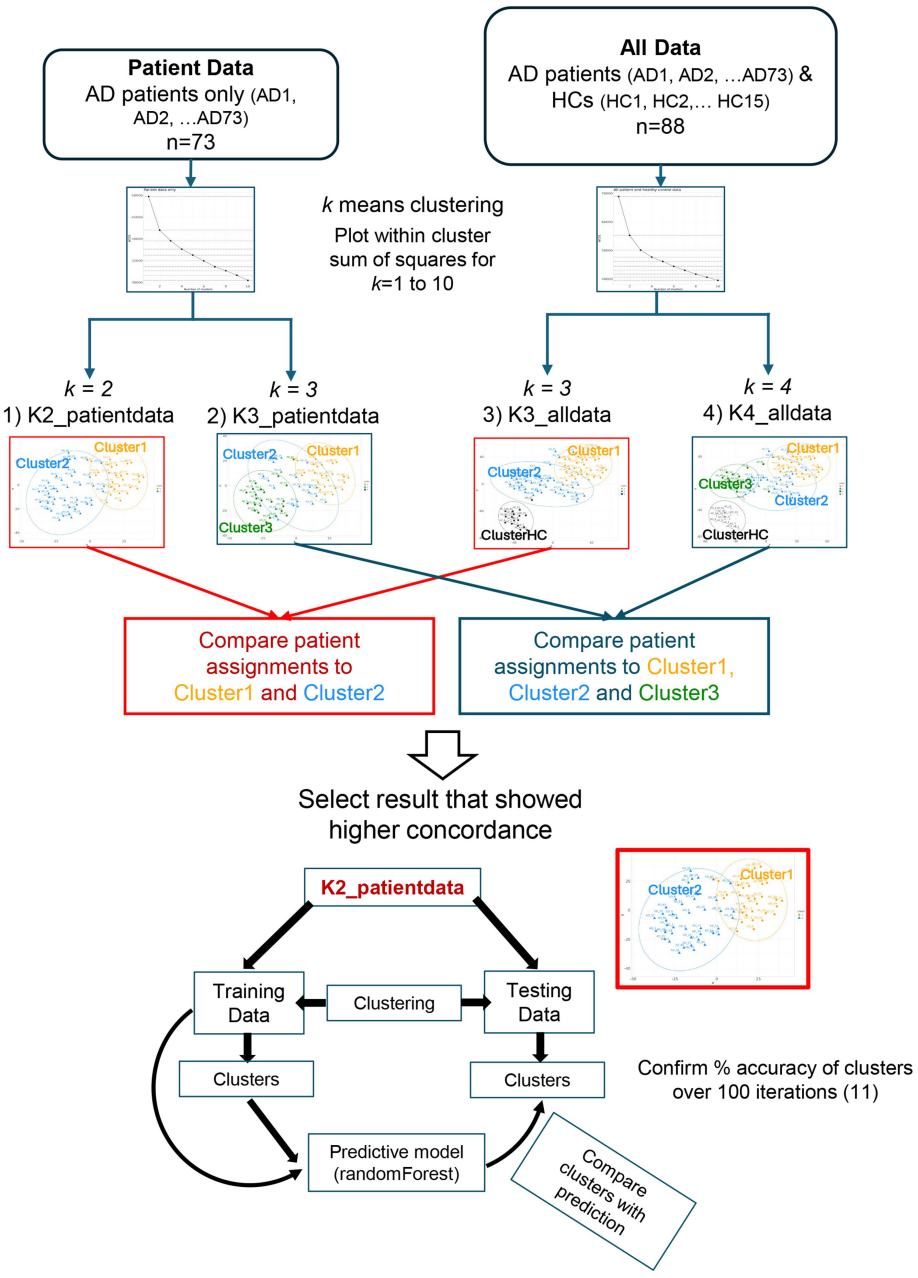
Methodology for assessing cluster stability and reproducibility. Flowchart outlining the steps undertaken to assess cluster stability and reproducibility. AD, atopic dermatitis; HC, healthy control.

Patient clusters were generated using the *k*-means clustering algorithm applied to serum proteomic data, as we had previously validated the accuracy of this algorithm using a bootstrapping method (11). To visualize cluster behavior, the within-cluster sum of squares (WCSS) for 1 to 10 clusters (*k* = 1 through 10) was first obtained using the *k*-means function in the R package: stats (16). Based on this, an optimal number of clusters was selected and assessed for stability and reproducibility. Refer to the flowchart shown in Figure 1 for a schematic representation of the overall cluster determination strategy.

#### 2.3.1 Cluster stability

Stability of clusters was assessed by including or excluding HC data from the clustering analysis to perturb the dataset. *K*-means clustering was performed separately for patient data only (*n* = 73) and for the combined patient and HC dataset (*n* = 88). Based on scree plots of the WCSS on patient data, we postulated that either *k* = 2 or *k* = 3 could represent a plausible number of patient clusters. Since HCs were expected to form a separate cluster, we assessed clustering outcomes using *k* = 3 (two patient clusters + one HC cluster) or *k* = 4 (three patient clusters + one HC cluster) for all data (*n* = 88). For cluster numbers greater than three (in patient data) or greater than four (in all data), the reduction in WCSS became less significant and stabilized with each additional cluster. Based on this, *k*-means clustering was performed using the following parameters and data input:

1. K2_patientdata: *k* = 2, all patient data only (*n* = 73).
2. K3_patientdata: *k* = 3, all patient data only (*n* = 73).
3. K3_alldata: *k* = 3, all patient and HC data (*n* = 88).
4. K4_alldata: *k* = 4, all patient and HC data (*n* = 88).

**Figure 2:**
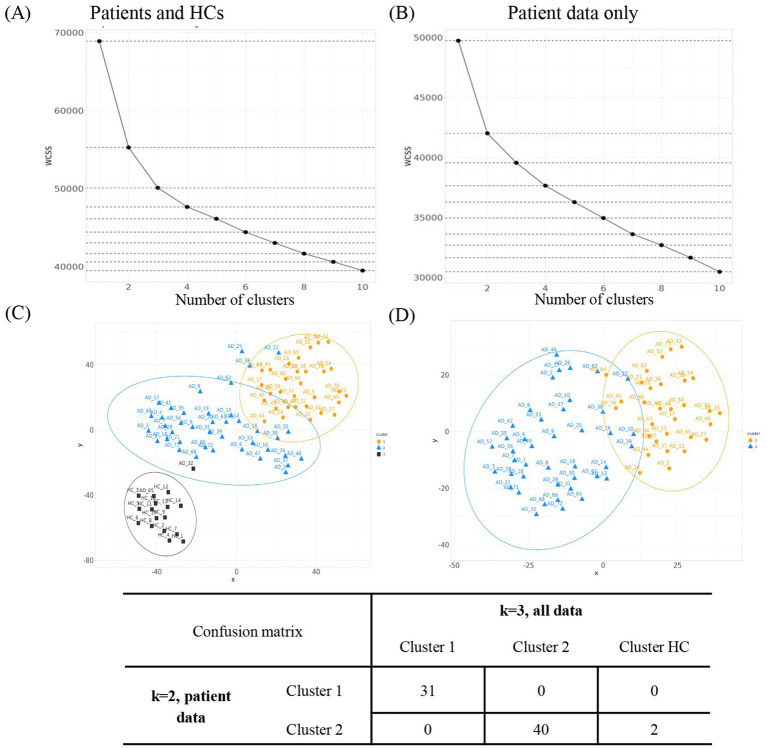
Evaluation of the optimal number of clusters. WCSS for *k* = 1 to *k* = 10 was assessed for k-means clustering on **(A)** all patients including HCs (K3_alldata) or on **(B)** patient data only (K2_patientdata). **(C)** *t*-SNE projection of the high-dimensional proteomic data into a two-dimensional space for clusters derived using K3_alldata. **(D)** *t*-SNE projections for clusters derived using K2_patientdata. Orange = cluster 1; blue = cluster 2; black = cluster 3. AD, atopic dermatitis; HC, healthy control; *t*-SNE, *t*-distributed stochastic neighbor embedding; WCSS, within-cluster sum of squares.

The outcomes of K2_patientdata were compared to K3_alldata, and the results are shown in Figure 2. The assignment of each patient into one of the two clusters in K2_patientdata was compared to that in K3_alldata using a confusion matrix (R package: caret). The same comparison was performed between K3_patientdata and K4_alldata (Supplementary Figure S1).

#### 2.3.2 Cluster reproducibility

To assess the reproducibility of the two patient clusters, we used a method previously published by our group. First, the samples were split into training and testing sets. Second, the same training and testing sets were subjected to *k*-means clustering with *k* = 2. Third, a cluster prediction rule was established using a random forest model (R package: randomForest), and the percent accuracy for the training set was calculated. This prediction rule was then applied to predict membership for each sample in the testing set, and the percent accuracy was calculated. Finally, the entire procedure was repeated 100 times for each scenario, and percent accuracy values for all 100 iterations were calculated for both the training and testing sets.

#### 2.3.3 Cluster visualization

To visualize the clusters, data dimensionality was first reduced by *t*-distributed stochastic neighbor embedding (*t*-SNE) analysis using the R package: *t*-SNE (17). The *t*-SNE projections were then used to visualize the clusters and evaluate their distinctiveness. The 95% confidence intervals for each cluster were drawn using the R package: ggplot2 (18).

### 2.4 Proteomic data visualization and differential expression analysis

Scaled proteomic data were visualized using a heatmap to evaluate overall protein expression in patients with AD and HCs (R package: ComplexHeatmap). Differential protein expression was performed using linear models for microarray data (R package: limma) (19), accounting for age and sex as covariates. An empirical Bayes step was applied to moderate the residual variances by borrowing strength between features in high-dimensional data (19). After fitting the model, a mean–variance plot for the full dataset was evaluated to determine whether assumptions were appropriate. Adjusted *p*-values were calculated using the Benjamini and Hochberg (BH) method to control the false discovery rate across markers (20). A fold-change threshold of >1.2 or <−1.2 (log₂ scale) and an adjusted *p*-value of <0.05 were applied to identify meaningful changes in expression levels. A volcano plot was generated to visualize log_2_ fold change for each protein, including negative log_10_-transformed adjusted *p*-values (R package: EnhancedVolcano) (21).

### 2.5 Predictive model generation using clinical data

All clinical data (described in Methods Section 2.1) were used as input into a random forest algorithm to generate a model that can best predict the proteomic data-derived clusters (R package: randomForest) (22). The top clinical data predictors of cluster membership were evaluated by mean decreases in accuracy and the Gini index, both used as measures of variable importance.

### 2.6 Weighted gene co-expression network analysis and hub network visualization

Weighted gene co-expression network analysis (WGCNA) was used to identify biologically functional modules of co-expressed proteins, as previously described (23) (R package: WGCNA) (24). To generate modules, a scale-free, weighted, signed network was assumed, and a soft adjacency matrix was computed using a threshold of 9—selected as the lowest power at which the scale-free topology model fit reached an *R*^2^ of 0.9. Refer to the Supplementary methods for module detection steps and specific parameters.

Within each module, all proteins are highly correlated; thus, modules are numerically represented by their first principal component, defined as the module eigenprotein (ME). The Pearson correlation between the ME and clinical and laboratory data was calculated and visualized as a heatmap. Adjusted *p*-values were calculated using the BH method to control the false discovery rate. A hub protein—defined as the protein with the highest intramodular connectivity (kME) for each module—was identified using the chooseTopHubInEachModule function.

Hub protein network graphs for the three largest modules—MEturquoise, MEbrown, and MEblue—were generated using the following steps: (1) all differentially expressed (DE) proteins (from Results Section 3.3) were identified within each of the three modules; (2) the top 30 most strongly correlated proteins in each module hub were identified, regardless of whether they were detected as a DE protein; and (3) these proteins were overlaid to construct each module’s network graph. Edge lengths between nodes were determined using values from the adjacency matrix generated earlier in this section. Network graphs were plotted using the R package: igraph (25).

### 2.7 Pathway analysis

To characterize protein modules identified by WGCNA, two methods were used to perform pathway analyses: (1) gene set enrichment analysis (GSEA) and (2) overrepresentation analysis using a hypergeometric test (R package: org.Hs.eg.db and clusterProfiler) (26, 27). For GSEA, the Pearson correlation between each protein NPX value and ME was calculated and used as a rank score for each protein. For the overrepresentation analysis, all proteins assigned to each module were used as input to the hypergeometric test. The “universe” for the hypergeometric test was defined as all 1,248 proteins analyzed for this study. The Gene Ontology (GO) “biological process” class of terms was used as a reference for both pathway analysis methods. Adjusted *p*-values for pathway analyses were calculated using the BH method (20).

### 2.8 Other statistical tests

The Wilcoxon rank-sum test was used to compare continuous variables (such as disease scores, clinical laboratory measures, and demographic background information) between the identified clusters. Categorical variables were compared using the chi-squared test. In all analyses, *p* < 0.05 was considered statistically significant. All analyses were performed using R software (version 4.3.2).

## 3 Results

### 3.1 Patient demographics and clinical characteristics

This study analyzed serum samples from 73 Japanese patients with moderate-to-severe AD from the BREEZE-AD2 study, with a mean [standard deviation (SD)] age of 36.3 (10.8) years and a mean (SD) BMI of 24.0 (4.4) kg/m^2^. The mean (SD) EASI score in this cohort was 31.5 (12.1). Patient demographics and clinical characteristics are summarized in Table 1. The AD clinical scores used in this analysis are outlined in Supplementary Table S1. Age- and sex-matched HC subjects had a mean (SD) age of 36.2 (12.2) years and BMI of 22.9 (2.6) kg/m^2^.

**Table 1:** Demographic and clinical characteristics of Japanese patients with AD.

	Total (*N* = 73)	AD_HI (*N* = 31)	AD_LO (*N* = 42)
Age, years, mean (SD)	36.3 (10.8)	36.3 (11.3)	36.3 (10.5)
Sex, *n* (%)			
Male	39 (53.4)	21 (67.7)	18 (42.9)
Female	34 (46.6)	10 (32.3)	24 (57.1)
Age at diagnosis, years			
Mean (SD)	9.9 (11.2)	13.4 (11.8)	7.3 (10.1)^*^
<18, *n* (%)	55 (75.3)	17 (54.8)	38 (90.5)^**^
≥18 to <50, *n* (%)	18 (24.7)	14 (45.2)	4 (9.5)^**^
BMI, kg/m^2^, mean (SD)	24.0 (4.4)	23.6 (3.9)	24.4 (4.8)
EASI score, mean (SD)	31.5 (12.1)	37.7 (12.2)	26.9 (9.8)^***^
SCORAD score, mean (SD)	67.3 (11.6)	74.2 (12.2)	62.2 (8.2)^***^
BSA, %, mean (SD)	58.0 (20.8)	68.9 (19.0)	50.0 (18.5)^***^
Prior therapies, *n* (%)			
Systemic	31 (42.5)	14 (45.2)	17 (40.5)
Topical only	42 (57.5)	17 (54.8)	25 (59.5)
Topical calcineurin inhibitors	50 (68.5)	24 (77.4)	26 (61.9)
Cyclosporin	13 (17.8)	7 (22.6)	6 (14.3)
IgE, IU/mL, mean (SD)	5,530 (7,090)	7,760 (8,190)	3,890 (5,720)^*^
Aspartate aminotransferase, IU/L, mean (SD)	23.9 (8.4)	27.2 (7.4)	21.4 (8.3)^**^
Cystatin C, mg/L, mean (SD)	0.9 (0.1)	0.9 (0.1)	0.8 (0.1)^***^
eGFR, mL/min/1.73m^2^, mean (SD)	110 (13.4)	109 (12.3)	111 (14.3)
Itch NRS, mean (SD)	6.6 (2.0)	7.3 (2.0)	6.1 (1.8)^**^
Triglycerides, mg/dL, mean (SD)	114 (61.1)	115 (55.7)	114 (65.5)
	***N* = 70**	***N* = 30**	***N* = 40**
Eosinophil count, × 10^9^/L, mean (SD)	0.7 (0.6)	1.1 (0.8)	0.4 (0.2)^***^
Neutrophil count, × 10^9^/L, mean (SD)	4.2 (1.4)	4.7 (1.4)	3.8 (1.3)^**^

^*^p < 0.05, ^**^p ≤ 0.01, and ^***^p < 0.001 for the AD_HI versus AD_LO cluster.

AD, atopic dermatitis; BMI, body mass index; BSA, body surface area; EASI, Eczema Area and Severity Index; eGFR, estimated glomerular filtration rate; HI, high; Ig, immunoglobulin; LO, low; NRS, Numeric Rating Scale; SCORAD, SCORing Atopic Dermatitis; SD, standard deviation.

### 3.2 Cluster analysis suggests two stable and reproducible clusters

We had previously reported that *k*-means clustering could define two reproducible clusters within AD patients based on their circulatory protein profile—the “high inflammatory” cluster and the “low inflammatory” cluster (11). Building on this, we utilized the WCSS approach to visualize the optimal number of clusters within our current dataset (Figures 2A,B). We then assessed the stability of patient clusters by comparing clustering results with and without HC data. Based on prior knowledge, we expected two patient clusters. We therefore hypothesized that K3_alldata would produce the same patient clusters as K2_patientdata, with HC samples in the K3_alldata set segregating into a third cluster separate from the two patient clusters. When clusters for K2_patientdata and K3_alldata were compared, the two patient clusters derived from both analyses were largely similar, with only two “outlier” patients from cluster 2 clustering with the HC cluster in K3_alldata (labeled AD32 and AD65) (Figures 2C,D). Therefore, clustering based on the assumption of two patient clusters and the addition/removal of the HC data from the dataset did not lead to significant changes in patient cluster assignment, suggesting stable clusters.

K3_patientdata (three hypothetical patient clusters) and K4_alldata (three hypothetical patient clusters + one HC cluster) were similarly evaluated. One patient cluster appeared stable in this comparison, but clusters 2 and 3 contained different assignments when HC data were excluded from the dataset (Supplementary Figure S1). Based on these observations, we deemed K2_patientdata to produce the most stable patient clusters and used these for conducting further downstream analyses.

Next, the reproducibility of clusters was evaluated for K2_patientdata in 100 iterations using our proposed method. The median percent accuracy was 92% for the training set and 86% for the testing set, suggesting that the clusters were highly reproducible.

### 3.3 Two clusters have distinct protein expression profiles

**Figure 3:**
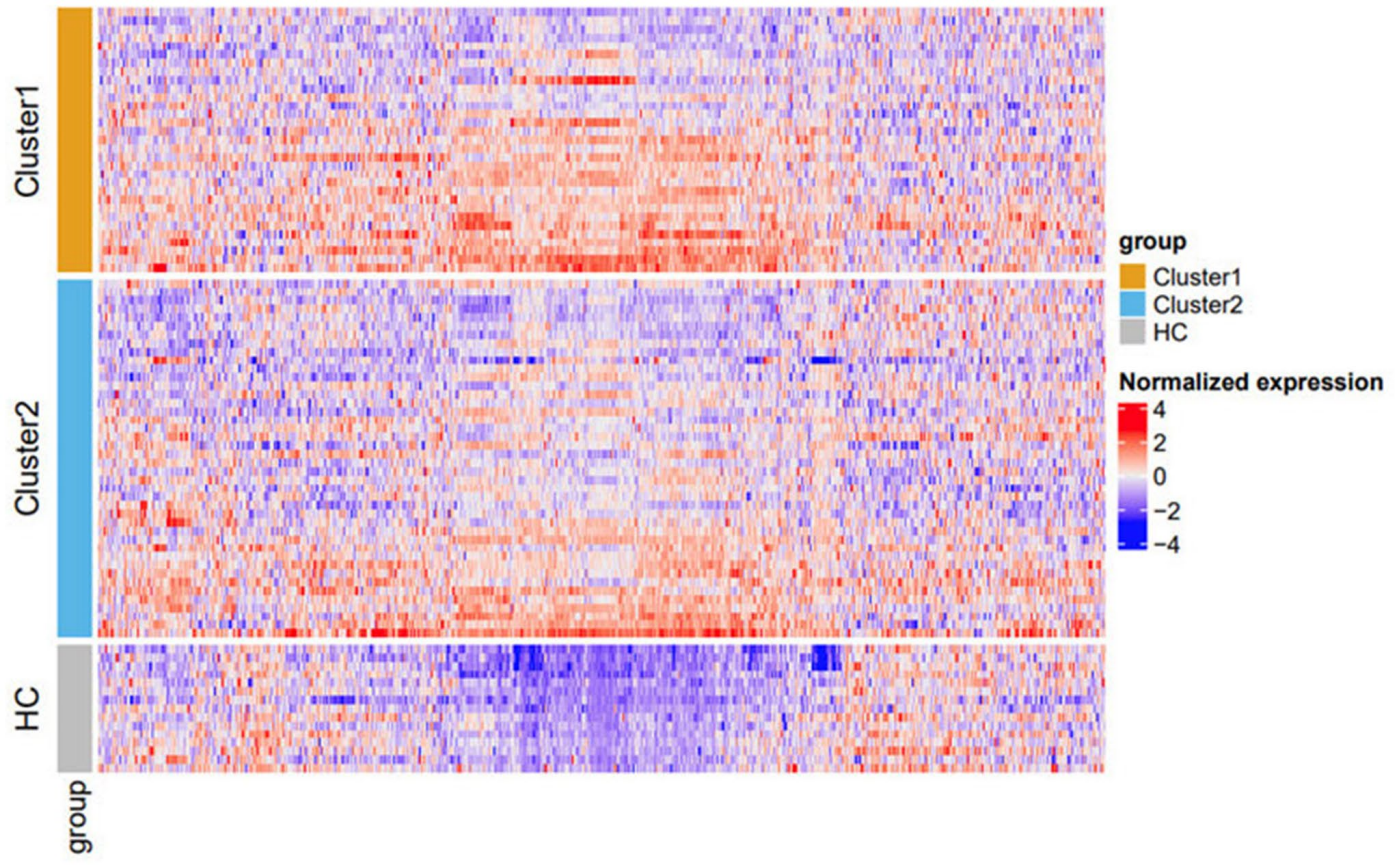
Heatmap of protein expression stratified by cluster. Cluster 1 (orange) and cluster 2 (blue). HCs are represented in gray. Within each subgroup, rows (samples) on the heatmap are sorted based on the *x*-value on the *t*-SNE plot (Figure 2C). Columns (proteins) are sorted using the default hierarchical clustering function in ComplexHeatmap. The color scheme is based on scaled and centralized protein expression data per marker across samples: red = higher expression; blue = lower expression. Differentially expressed proteins between the clusters are detailed in Supplementary Table S3. HC, healthy control; *t*-SNE, *t*-distributed stochastic neighbor embedding.

Protein expression profiles were evaluated from patient serum samples after a medication washout period, as specified in the clinical trial protocol. This controlled for the effects of prior medication, such as topical or oral steroid use, on protein expression, ensuring that heterogeneity observed in the profiles closely reflects actual biological differences in disease states. Based on these proteomic profiles, the K2_patientdata combination generated two clusters: cluster 1 [*n* = 31 (42%)] and cluster 2 [*n* = 42 (58%)]. The proteomic profile of both clusters and HCs was visualized using a heatmap to evaluate relative protein expression levels across the entire protein panel (Figure 3). HCs had visibly lower expression across much of the proteomic panel compared to the two patient clusters (Figure 3).

### 3.4 DE proteins in the two AD clusters

**Figure 4:**
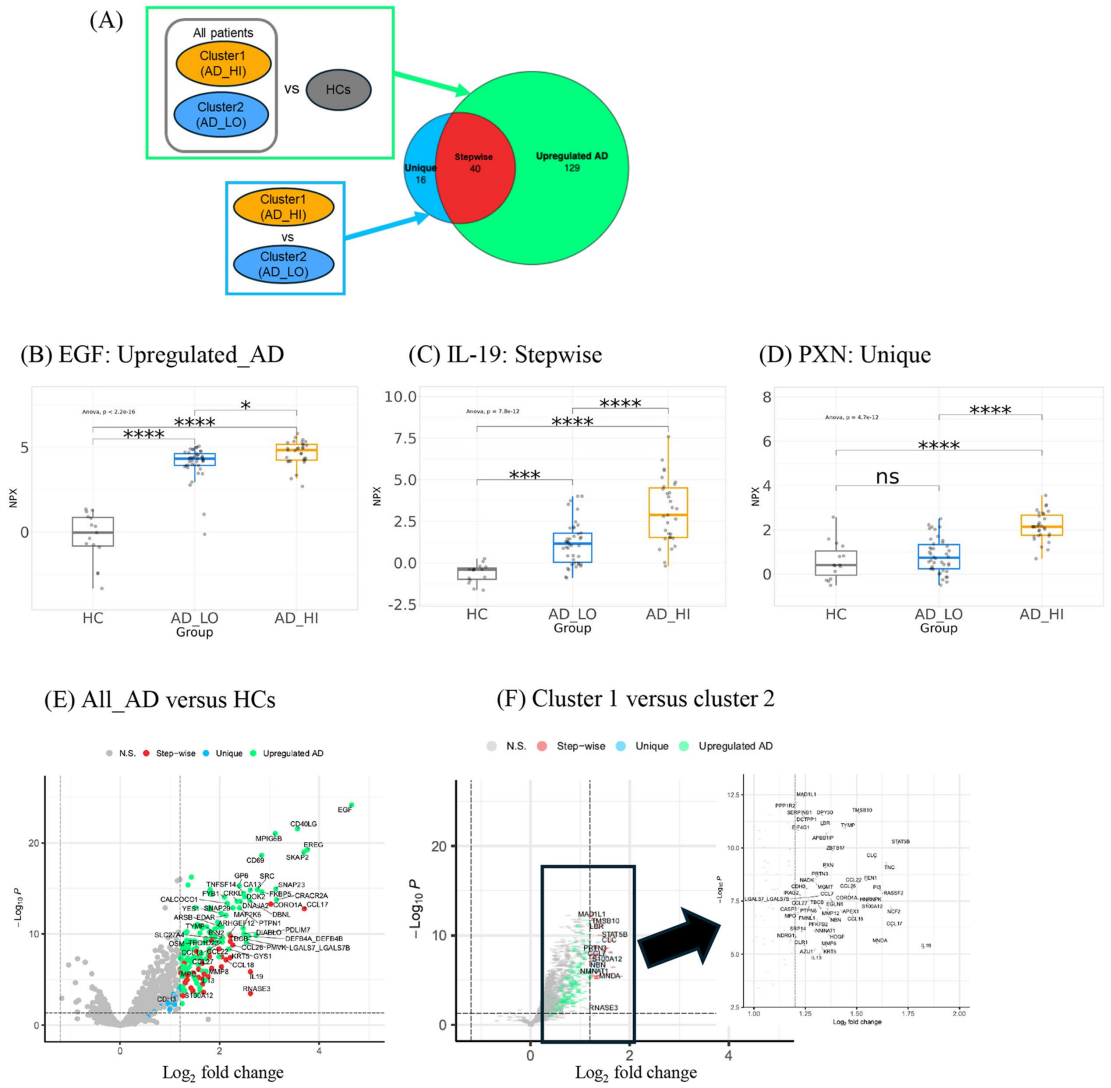
Differential expression analysis of proteins in clusters and HCs derives three groups of proteins with distinct trends in expression. **(A)** Description of comparison groups in two differential expression analyses performed. Respective results are shown in a Venn diagram illustrating the number and overlap of DE proteins detected. The large circle represents the 169 proteins upregulated in all AD versus HCs. The smaller circle represents the 56 proteins upregulated in cluster 1 over cluster 2. The three Venn diagram sections represent the three groups of DE proteins: Unique, Stepwise, and Upregulated AD. The 40 stepwise proteins represent the quantitative difference between clusters 1 and 2. **(B)** Expression patterns of representative proteins (largest fold change selected) in the **(C)** “Upregulated AD” group, **(D)** “Stepwise” group, and **(E)** “Unique” group. ^*^p < 0.05, ^**^p < 0.01, ^***^p < 0.001, and ^****^p < 0.0001. **(E)** Volcano plot shows upregulated proteins in ALL_AD (all patients with AD) over HCs. Only proteins associated with AD pathophysiology and all other proteins with log_2_FC >2 were labeled. **(F)** Volcano plot shows upregulated proteins in cluster 1 over cluster 2. The analyte log_2_ fold change is plotted on the *x*-axis, and the negative log10-transformed adjusted *p*-value is plotted on the *y*-axis. AD, atopic dermatitis; ANOVA, analysis of variance; DE, differentially expressed; EGF, epidermal growth factor; HC, healthy control; HI, high; IL, interleukin; LO, low; NPX, normalized protein expression; ns, not significant; PXN, paxillin.

To further characterize differences in the protein expression profiles between the two patient clusters, a linear mixed model was used to evaluate differential protein expression, adjusting for age and sex as covariates. Two comparisons were made: (1) all patients versus HCs and (2) cluster 1 versus cluster 2. In these comparisons, 169 and 56 DE proteins (log_2_ fold change >1.2) were detected in all patients versus HCs and in cluster 1 versus cluster 2, respectively (Figure 4A). There were no differentially downregulated proteins that reached the fold-change cutoff and statistical significance in both comparisons.

The DE proteins could be categorized into three groups: (1) “Upregulated AD”—proteins upregulated in all AD patients; (2) “Stepwise”—proteins with the lowest expression in HCs, higher expression in cluster 2, and highest expression in cluster 1; and (3) “Unique”—proteins upregulated in cluster 1 but not cluster 2. Representative proteins with the largest fold change from each of the three groups are shown in Figures 4B–D.

The proteins with the largest fold change in the “Upregulated AD” group included epidermal growth factor (EGF), followed by epiregulin (EREG) and cluster of differentiation 40 ligand (CD40LG) (Figure 4E). Notably, CD40LG and CD69 are markers of activated T cells. The 40 “Stepwise” proteins represent the quantitative difference between clusters 1 and 2, as illustrated by the overlap in the Venn diagram in Figure 4A. The top proteins with the largest log_2_ fold change in this group included IL-19, STAT5B, CCL17, S100A12, and CCL22—inflammatory mediators known to be associated with AD inflammation and eosinophilia (Figure 4F). Other cytokines and chemokines characteristic of AD pathology, such as pulmonary and activation-regulated chemokine (PARC/CCL18), eotaxin-3/CCL26, macrophage-derived chemokine (MDC/CCL22), cutaneous T-cell-attracting chemokine (CTACK/CCL27), and IL-13, were also among the 40 “Stepwise” proteins.

The 16 “Unique” proteins upregulated only in cluster 1 may represent biological features specific to this subgroup (Figure 4F). These included proteins involved in cell proliferation and migration (PXN, PTPN6, CDH3, FMNL1, and HDGF), cell stress (NCF2, EGLN1, and NDRG1), cellular metabolism (HNRNPK, NMNAT1, and SRP14), and DNA repair (MGMT, APEX1, and NBN). A full list of the three groups of DE proteins and their fold-change values is available in Supplementary Tables S2, S3. Based on these DE protein profiles, cluster 1 was labeled the high-inflammatory cluster (AD_HI), and cluster 2 was labeled the low-inflammatory cluster (AD_LO).

### 3.5 Characterization of protein modules associated with clusters, markers of disease severity, and metabolic function

**Figure 5:**
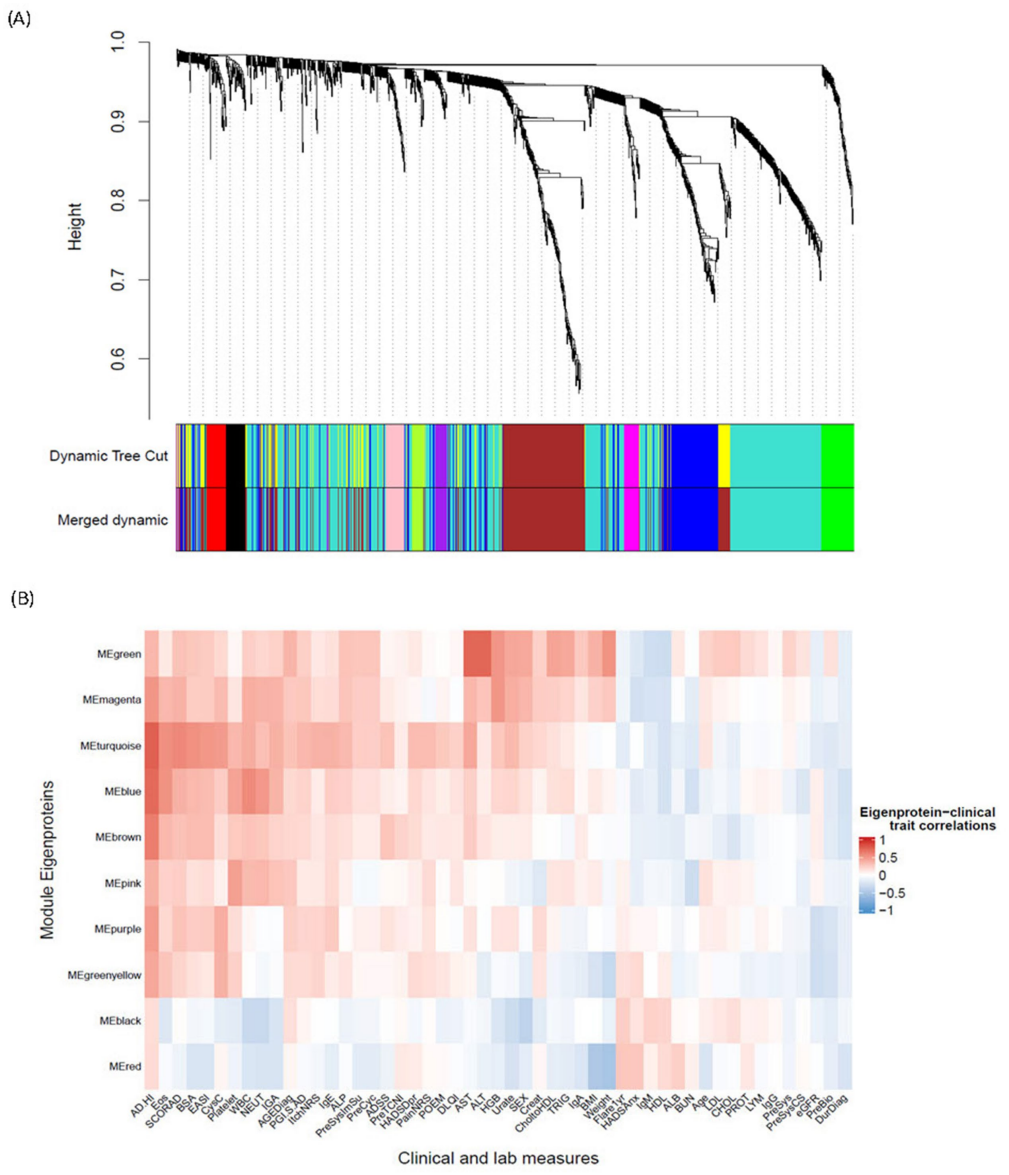
WGCNA derivation of protein modules and their association with endotypes and clinical measures. **(A)** Cluster dendrogram illustrates 1,248 protein analytes clustered into 12 initial modules (represented in “Dynamic Tree Cut”) and 11 final modules (represented in “Merged Dynamic”) identified via a WGCNA algorithm. The row “Merged Dynamic” shows the 10 modules of interest used in subsequent analyses, plus one module (MEgrey) comprising only one protein (FCRL3). **(B)** Heatmap shows the correlation between the ME of each of the final 10 modules of interest and a clinical trait. The color scheme is based on the Pearson correlation value between the ME and the clinical trait value: red = positive correlation; blue = negative correlation. AD, atopic dermatitis; ADSS, Atopic Dermatitis Symptom Score; AGEDiag, age at diagnosis; ALB, albumin; ALP, alkaline phosphatase; ALT, alanine aminotransferase; AST, aspartate aminotransferase; BMI, body mass index; BSA, body surface area; BUN, blood urea nitrogen; CHOL, cholesterol; CholtoHDL, cholesterol-to-HDL ratio; Creat, creatinine; CysC, cystatin C; DLQI, Dermatology Life Quality Index; DurDiag, duration since first diagnosis; EASI, Eczema Area and Severity Index; eGFR, estimated glomerular filtration rate; Eos, eosinophils; FCRL3, fragment crystallizable receptor-like protein 3; Flare1yr, flare in the past year; HADSAnx, Hospital Anxiety and Depression Scale—Anxiety; HADSDpr, Hospital Anxiety and Depression Scale—Depression; HDL, high-density lipoprotein; HGB, hemoglobin; HI, high; Ig, immunoglobulin; IGA, Investigator Global Assessment; LDL, low-density lipoprotein; LYM, lymphocytes; ME, module eigenprotein; NEUT, neutrophils; NRS, Numeric Rating Scale; PGI.S, Patient Global Impression of Severity; POEM, Patient-Oriented Eczema Measure; PreBio, prior biologic; PreCyc, prior cyclosporin; PreSys, prior systemic therapy; PreSysCS, prior systemic corticosteroid; PreSysImSu, prior systemic immune suppressant; PreTCNI, prior topical calcineurin inhibitor; PROT, protein; SCORAD, SCORing Atopic Dermatitis; TRIG, triglycerides; WBC, white blood cell; WGCNA, weighted gene co-expression network analysis.

To further characterize differences in protein expression profiles between the two clusters and how these differences correlate with disease measures, we used WGCNA—an unsupervised algorithm that identifies networks (modules) of highly correlated genes or proteins and reduces dimensionality in the proteomic data. The WGCNA algorithm clustered 1,248 protein analytes into 10 final modules of interest (Figure 5A). The number of proteins comprising each module ranged from 22 to 536 (Table 2). The hub protein for each module (the protein most strongly correlated to all other proteins within that module) is shown in Table 2. Each module’s correlation to clinical data and cluster assignment was also evaluated, and the correlation coefficient was visualized on a heatmap (Figure 5B). Coefficients and adjusted *p-*values for all correlations can be found in the source data. The modules—MEturquoise, MEbrown, and MEblue—were most strongly correlated with AD disease scores, such as SCORing Atopic Dermatitis (SCORAD), body surface area (BSA), and EASI, indicating that the biological pathways represented by these modules are associated with disease severity (Figure 5B). Notably, these three disease modules were also found be the largest. In addition to these, five more modules (MEgreen, MEmagenta, MEpink, MEpurple, and MEgreenyellow) were most strongly and significantly associated with the AD_HI cluster (Pearson coefficient >0.3, adj. *p* < 0.05) (source data file). These five modules were strongly correlated with liver enzyme levels [aspartate aminotransferase (AST), alanine aminotransferase (ALT)], white cell counts, triglycerides, BMI, and weight, indicating heterogeneity in the biology observed in the AD_HI cluster. This observation is also consistent with previous findings showing that upregulated proteins in the AD_HI cluster comprise both canonical and non-canonical AD protein biomarkers (11). This cluster-module-clinical trait correlation approach further characterized the difference in molecular profile between the AD_HI and AD_LO clusters. Subsequent analysis focused on the relationship between the three disease-associated modules and the DE proteins described in Section 3.4.

**Table 2:** Summary characteristics of WGCNA-derived protein modules, including the hub protein, protein biomarker most correlated with the eigenprotein, module size, and clinical data most correlated with the eigenprotein.

Protein module	Hub protein	Size	Clinical parameter
Turquoise	DPY30	536	Disease severity (WBC count, neutrophils, eosinophils, EASI score, BSA, SCORAD score, and cystatin C)
Brown	CC2D1A	290	
Blue	PXN	178	
Green	ADH4	58	AST, ALT, body weight, cholesterol, triglycerides, and hemoglobin
Black	CNTN1	37	Sex, WBC count, and neutrophils
Red	BCAN	37	BMI and body weight
Pink	VEGFC	34	Platelet count
Magenta	DNPH1	28	Hemoglobin, urate, and WBC count
Purple	CD93	27	Cystatin C
Green-yellow	NBL1	22	Cystatin C and body weight

ALT, alanine aminotransferase; AST, aspartate aminotransferase; BMI, body mass index; BSA, body surface area; EASI, Eczema Area and Severity Index; SCORAD, SCORing Atopic Dermatitis; WBC, white blood cell; WGCNA, weighted gene co-expression network analysis.

**Figure 6:**
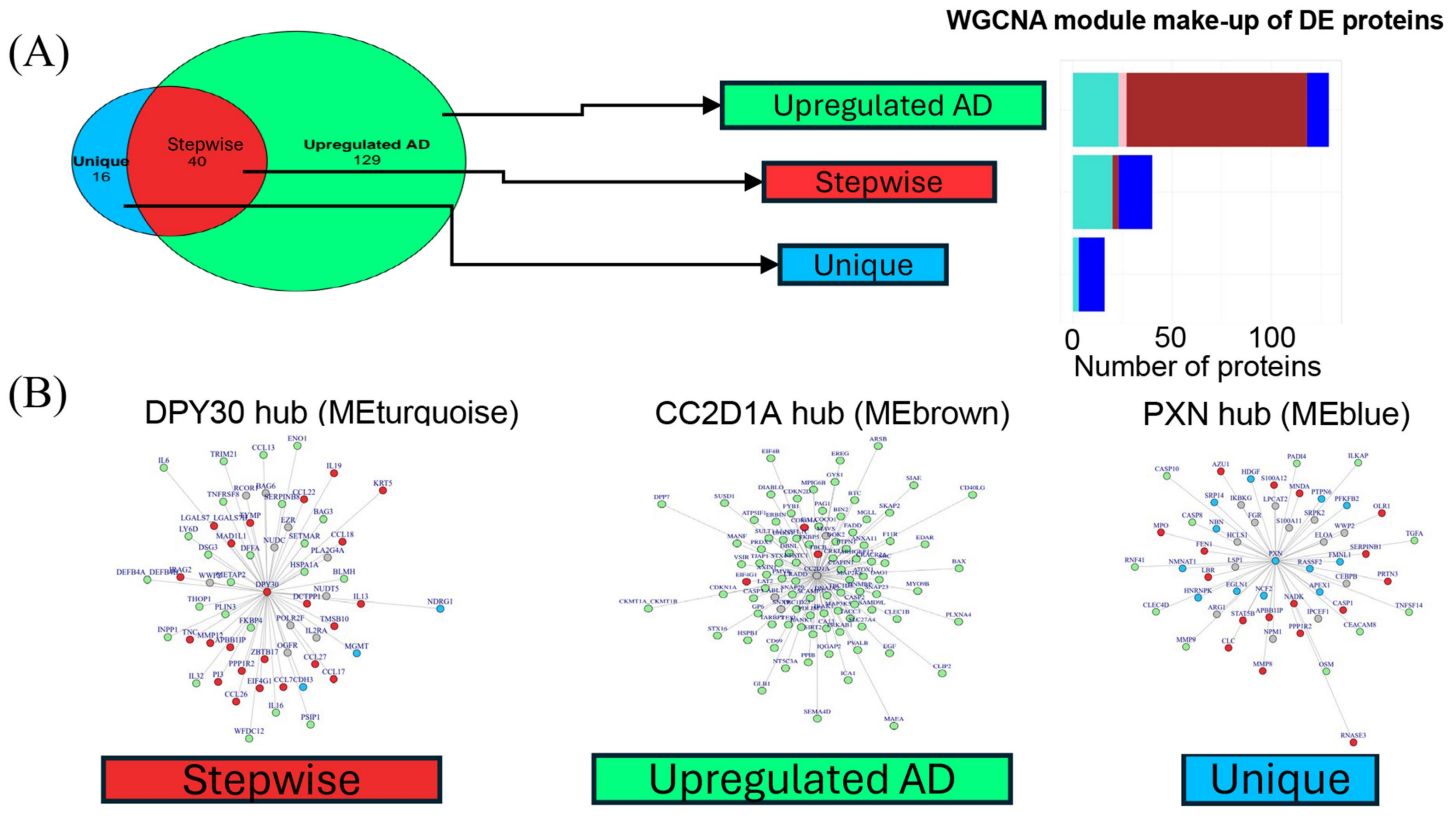
Hub network visualization of disease module proteins. **(A)** Bar graph shows the three groups of DE proteins and the WGCNA protein module they map to. Bar colors correspond to the WGCNA protein module colors. All DE proteins belonged to one of four main WGCNA protein modules: turquoise, pink, brown, and blue. **(B)** Network graphs for the three largest WGCNA protein modules. A graph was generated for each hub protein. Nodes represent the most correlated proteins within each module. Edge length reflects connectivity strength between nodes—shorter edges indicate stronger connectivity. Nodes are color-coded by the protein group assigned in the differential expression analysis: “Upregulated AD” (green), “Stepwise” (red), or “Unique” (blue). Gray nodes represent proteins that were not DE but were strongly connected components in the protein hubs. DE proteins were typically closely connected to the hub, resulting in few gray nodes. AD, atopic dermatitis; DE, differentially expressed; HC, healthy control; HI, high; LO, low; ME, module eigenprotein; WGCNA, weighted gene co-expression network analysis.

All DE proteins described in Section 3.4 were grouped within the three disease modules, MEturquoise (46 proteins), MEbrown (94 proteins), and MEblue (41 proteins), as well as MEpink (4 proteins). The WGCNA protein module makeup of the three groups of DE proteins is shown in Figure 6A. DE proteins within the disease modules and their connectivity to the hub protein were visualized on a network graph for each hub. Known inflammatory mediators of AD inflammation in the “Stepwise” group—CCL17, CCL18, CCL26, CCL22, CCL27, IL-19, and IL-13—are closely connected to the dpy-30 histone methyltransferase complex regulatory subunit (DPY30; MEturquoise) hub, as shown in red in Figure 6B. Other pro-inflammatory mediators, such as IL-6 and IL2RA, were also located in this hub, suggesting that this module represents an AD-specific inflammatory module correlated with the AD_HI and AD_LO clusters, but to a larger extent with the AD_HI cluster (“Stepwise” trend).

MEbrown contains the hub protein CC2D1A (coiled-coil and C2 domain containing 1A). The majority of the “Upregulated AD” proteins (green nodes) are grouped in this module (Figure 6A). Of the 16 total “Unique” proteins (blue nodes) from the differential expression analysis, 13 were in the MEblue module connected to paxillin (PXN)—the hub protein for this module (Figure 6B).

Pathway analysis was performed to understand the protein makeup and biological function of each protein module. The full results of the two analyses are available in the source data, and a summary is provided in Table 3. Overall, the results suggest that the three major modules (MEturquoise, MEblue, and MEbrown) were enriched for pathways in cytokine signaling; adaptive immune response; protein, nucleotide, and cellular metabolism; and cell cycle regulation (Table 3). MEgreen, which was strongly correlated with liver enzymes and body weight, was enriched in proteins involved in organic and amino acid metabolism.

**Table 3:** Summary of pathway enrichment analysis (GOBP) results for each protein module as evaluated by GSEA or overrepresentation by hypergeometric analysis.

Protein module	Hypergeometric analysis	GSEA
Turquoise	Adaptive immune response, regulation of development, and T-cell differentiation	NA
Brown	Intracellular transport, organelle organization, and protein catabolic process	Cell adhesion, system development, and neurogenesis
Blue	Regulation of nucleotide metabolism and cellular metabolism	Organic cyclic compound, nitrogen compound metabolism, intracellular receptor signaling, and cell cycle
Green	Carboxylic acid, oxoacid, organic acid, and amino acid metabolism	Carboxylic acid, organic acid, metabolism, and catabolism
Black	Axonal and neuronal guidance, cell morphogenesis, and cell adhesion	Organic cyclic compound, nitrogen compound, cellular aromatic compound metabolism, and cell cycle
Red	NA	Cell adhesion
Pink	Cell surface receptor signaling and cell motility and migration	Multicellular organismal process, system development, cell adhesion, and organism development
Magenta	NA	Nucleic acid and RNA metabolism
Purple	NA	Immune system process, positive regulation of cellular process, locomotion, and taxis
Green–yellow	Negative regulation of signal transduction and serine/threonine kinase pathway	NA

A textual summary of the top GOBP pathway hits, with BH-adjusted *p* < 0.05 shown.

BH, Benjamin and Hochberg; GOBP, gene ontology biological processes; GSEA, gene set enrichment analysis; NA, not available (no result obtained from analysis); RNA, ribonucleic acid.

### 3.6 Association of the AD_HI endotype with disease severity scores and routine laboratory measures

**Figure 7:**
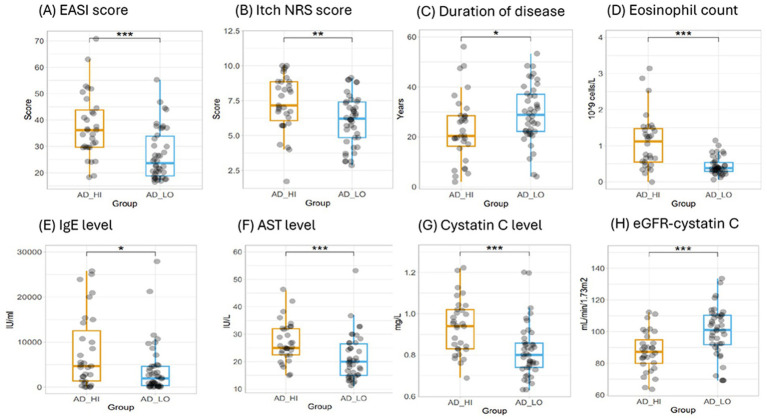
Evaluation of clinical and laboratory measures for the AD_HI and AD_LO endotypes. **(A)** EASI score, **(B)** Itch NRS score, **(C)** duration of disease, **(D)** eosinophil count, **(E)** IgE levels, **(F)** AST levels, **(G)** cystatin C levels, and **(H)** eGFR calculated using cystatin C. *p*-values were calculated using the Wilcoxon rank-sum test. ^*^p < 0.05, ^**^p < 0.01, and ^***^p < 0.001. AD, atopic dermatitis; AST, aspartate aminotransferase; EASI, Eczema Area and Severity Index; eGFR, estimated glomerular filtration rate; HI, high; Ig, immunoglobulin; LO, low; NRS, Numeric Rating Scale.

Finally, we evaluated all clinical (non-proteomic) data in a predictive model to objectively identify any potential clinical predictors for the two clusters. Eosinophil count was the strongest predictor for the clusters in this study, with all patients in AD_LO having a circulating eosinophil blood cell count ≤1.15 × 10^9^ cells/L (Figure 7). Eosinophil count was followed by EASI score, cystatin C level, age at AD diagnosis, and baseline Itch Numeric Rating Scale (NRS) score as the most predictive parameters (Supplementary Figure S2).

Based on the ranking of importance within the predictive model, selected clinical and laboratory measures representative of a range of physiological functions were assessed to confirm any statistically significant differences in mean values between the AD_HI and AD_LO clusters. The AD_HI cluster was associated with a higher mean (SD) eosinophil count [1.1 (0.8) vs. 0.4 (0.2) × 10^9^/L; *p* < 0.001], neutrophil count [4.7 (1.4) vs. 3.8 (1.3) × 10^9^/L; *p* < 0.01], and immunoglobulin E level [7,760 (8,190) vs. 3,890 (5,720) IU/mL; *p* < 0.05] compared with the AD_LO endotype (Table 1). The AD_HI cluster was also associated with higher levels of AST and cystatin C, suggesting a general hepatic burden and possible subclinical renal dysfunction. No significant differences were observed in estimated glomerular filtration rate (eGFR; calculated using serum creatinine) or triglycerides between AD_HI and AD_LO clusters; however, AD_HI was associated with a significantly lower mean eGFR when calculated using cystatin C values as previously described (28) [87.6 (12.7) vs. 99.9 (15.5) mL/min/1.73 m^2^; *p* < 0.001]. The AD_HI cluster was associated with a higher mean (SD) EASI score [37.7 (12.2) vs. 26.9 (9.8); *p* < 0.001], SCORAD score [74.2 (12.2) vs. 62.2 (8.2); *p* < 0.001], and Itch NRS score [7.3 (2.0) vs. 6.1 (1.8); *p* = 0.01], while the AD_LO cluster was associated with an earlier AD diagnosis (*p* = 0.02) (Table 1). Box plots showing data by cluster for each of the above parameters are provided in Figure 7; Supplementary Figure S3.

## 4 Discussion

To the best of our knowledge, this is the first comprehensive molecular endotyping study of AD in a Japanese-only patient population using clinical trial data and a broad panel of immune- and non-immune-related protein analytes. Although biomarker-defined AD endotypes have been increasingly reported, the majority of studies focus on European populations, with limited representation of Asian cohorts. Expanding research to include diverse ethnic groups is warranted to advance knowledge in this field (29).

We previously established that *k*-means clustering with *k* = 2 yielded reproducible clusters in a mixed cohort of AD patients, including Caucasian, African American, and Asian individuals (11). These two clusters differed in their inflammatory profiles, with one cluster exhibiting elevated inflammation levels and increased disease severity. In the current Japanese cohort, we identified two stable and reproducible clusters representing putative molecular endotypes of AD, distinguished by inflammatory profile. We designated these as AD_HI (high inflammatory) and AD_LO (low inflammatory) clusters.

We conducted DE analysis followed by WGCNA to investigate protein signatures associated with the defined clusters. The DE analysis revealed a quantitative difference between the two patient clusters, highlighted by a group of “Stepwise” proteins. This difference is characterized by cytokines and chemokines commonly associated with AD, including IL-13, IL-19, TARC (CCL17), PARC (CCL18), eotaxin-3 (CCL26), CCL22, and CCL27. Previous studies have shown that these biomarkers correlate with disease severity in pediatric and adult AD cases (30–35).

Notably, STAT5B over-expression was evident in all AD patients and the AD_HI cluster. STAT5B acts downstream of JAK1, a gene in which gain-of-function mutations reportedly cause hypereosinophilic syndrome (36), and it is the target of the approved AD therapeutic agent baricitinib. High STAT5B expression in the AD_HI cluster aligns with the elevated eosinophil counts characteristic of this phenotype. Our analysis suggests that the level of inflammation, as reflected by these “Stepwise” circulating proteins, varies quantitatively within the AD patient population, and the magnitude of this variation may have clinical implications.

In Japan, TARC is an approved clinical biomarker for monitoring disease activity in AD patients, with a reference value of <450 pg/mL considered normal in healthy adults. Our results suggest that elevated TARC levels may mark a distinct disease state, potentially offering additional diagnostic or prognostic value. Further validation analyses are needed to understand the clinical utility of our identified clusters and, subsequently, to define higher TARC (and possibly a combination of other inflammatory mediators) concentrations that may have clinical utility.

In addition to quantitative differences seen in the canonical inflammatory mediators, qualitative differences were observed, represented by the “Upregulated AD” and “Unique” proteins. These proteins are involved in diverse biological processes, including skin barrier function, cell proliferation and migration, metabolism, and DNA repair. The selective upregulation of the “Unique” proteins in the AD_HI cluster suggests there may be a distinct biological component in this subgroup that is absent in AD_LO, indicating that these clusters may reflect different disease states. This finding also suggests that dysregulation of pathways other than cytokine-mediated inflammation could contribute to disease severity.

To further elucidate the protein signatures associated with the clusters, we performed WGCNA. This analysis identified three protein networks (modules) that were strongly correlated with the AD_HI cluster and with measures of disease severity, including EASI, SCORAD, and eosinophil count. Notably, all “Stepwise” canonical AD inflammatory mediators were closely connected to the largest module, reinforcing their key role in AD pathophysiology. The hub protein of this module, DPY30, is an integral core component of the SET1/MLL family of H3K4 methyltransferases. This complex regulates the cell cycle and plays an important role in the proliferation and differentiation of human hematopoietic progenitor cells (37). DPY30 itself was also identified as one of the “Stepwise” proteins. While its role in AD has not been directly studied, DPY30’s function in epigenetic regulation, particularly histone H3K4 methylation, suggests it may influence immune cell differentiation and cytokine expression relevant to AD pathogenesis and other inflammatory disorders. DPY30 has been implicated in tumor-associated inflammation and showed correlations with tumor grade and immune-related gene activation in colorectal cancer, as well as immune cell infiltration in esophageal cancer (38, 39).

Although not significantly upregulated in our DE analyses, other pro-inflammatory cytokines such as IL2RA and IL-6 were also closely connected within the DPY30 hub. This indicates that the upregulation of type 2 inflammation in AD may be accompanied by other inflammatory axes. CC2D1A, another hub protein identified in a disease-associated module, functions as a transcriptional repressor in neuronal cells and has been linked to autism spectrum disorder, intellectual disability, and depression (40–42). While the significance of CC2D1A upregulation in AD is unclear, pathway analyses of the module proteins revealed involvement in processes including intracellular signaling, protein and nucleotide metabolism and transport, and cell adhesion. These pathways may be activated in response to external insults through the skin barrier, leading to enhanced innate cell activation and increased signaling at the innate-adaptive cell interface. PXN, the hub gene of the third disease module, is a focal adhesion protein involved in mediating intracellular signaling. Interactions between alpha-4 integrin and PXN have been used as targets to inhibit T-cell homing to sites of inflammation (43). The upregulation of PXN—along with other T-cell markers such as CD40LG and CD69—is particularly relevant given that AD is a T-cell-driven disease. PXN has also been implicated in several inflammatory and immune-related diseases, including rheumatoid arthritis and inflammatory bowel disease, as well as tumor-associated inflammation (44).

Aside from the three main disease modules, a broad evaluation of clusters to module to clinical trait correlations shows that the AD_HI cluster is most strongly correlated with many protein modules that are also linked to clinical traits (liver enzymes, weight, and BMI) beyond AD disease severity. These non-disease modules contained cell adhesion and carboxylic and organic acid metabolism processes. The specific role of these pathways in AD disease etiology remains to be elucidated.

We found key differences in clinical characteristics between the two clusters that may have direct implications for clinical practice. For instance, all patients in the AD_LO cluster had eosinophil counts below 1.15 × 10^9^/L. Given the normal adult reference range for circulating absolute eosinophil counts is 0.03–0.35 × 10^9^/L, our results indicate that eosinophilia is evident in a subset of the AD_LO cluster and is even more pronounced in the AD_HI cluster. Additionally, significantly higher EASI and SCORAD scores were seen in AD_HI compared to AD_LO, with mean differences of 10.8 and 12.0, respectively. Notably, 29 of the 31 patients in the AD_HI cluster were classified as having severe to very severe disease based on clinical definitions, with the remaining two patients classified as having moderate disease (EASI score ≤21). Finally, approximately 90% of patients in the AD_LO cluster were diagnosed before 18 years of age. Age of onset is an important clinical characteristic in AD, and a recent study showed that pediatric- and adult-onset AD exhibit distinct inflammatory profiles in skin and blood (45). Taken together, these findings suggest that a patient’s molecular endotype may be predicted using a combination of AD biomarkers (e.g., TARC), eosinophil count, EASI and SCORAD scores, and age of disease onset. This cross-validation of skin and blood measures also reinforces the clustering outcome and increases the relevance of the biology observed in our proposed endotypes. This further highlights the systemic nature of AD, where skin inflammation is reflected in the circulation.

Unexpectedly, we found that patients in the AD_HI cluster tended to have higher circulating cystatin C levels, which corresponded to significantly lower cystatin C-derived eGFR values. Circulating cystatin C is commonly used as a clinical measure of kidney function, with elevated levels potentially indicating subclinical renal impairment (46). Although research is limited, the severity of several inflammatory skin diseases, including atopic eczema, is weakly associated with chronic kidney disease (47). Elevated cystatin C in AD_HI could be attributed to the prolonged use of medication or the chronic inflammatory burden associated with AD; however, we were not able to draw such conclusions from our dataset. Cystatin C is a marker of inflammation in various disease states (48, 49), and elevated cystatin C is observed in patients with asthma, where it may act as an inflammatory mediator in the lungs (50). Future research examining the relationship between the duration of AD medication use and the inflammatory profile of the disease may provide new insights into the potential role of cystatin C as a biomarker of inflammation.

Our study differs from previous molecular endotyping research in that this cohort of patients underwent a strict topical and systemic AD treatment washout period prior to blood sample collection. As such, the circulating profile described in our cohort is not confounded by the immediate immunosuppressive effects of medication and could more closely reflect ongoing disease pathophysiology. However, prolonged use of immunosuppressive therapies is common in this patient population, and lasting effects on protein expression cannot be precluded. Circulating protein expression may also be influenced by external factors, such as skin infection with microbial pathogens and environmental irritants, given that a compromised skin barrier is a clinical feature of AD. Colonization with *Staphylococcus aureus* (*S. aureus*) is common in AD and is associated with a distinct phenotype marked by severe disease and impaired skin barrier function (51). In response to *S. aureus*, epithelial cell-derived cytokines such as TSLP and IL-33 further drive Th2 responses common in AD immunopathology (52). Environmental irritants, including detergents and pollutants, can also penetrate the compromised epidermal barrier in AD, leading to keratinocyte injury and localized inflammation. Together, external stimuli and compromised skin barrier function could affect the circulating protein profile observed in individuals with AD.

This study has limitations that should be considered when interpreting the findings. It included a relatively small sample size and captured only a single pre-treatment data point from Japanese patients enrolled in a clinical trial, which may limit the generalizability to the broader Japanese AD population. Additionally, our analysis was based on a manufacturer-defined limited protein panel comprising 1,248 protein analytes. Future studies using larger or unbiased proteomic approaches, such as mass spectrometry, may be well-suited to understanding non-canonical biological mechanisms contributing to AD heterogeneity. Despite these limitations, our findings support the reproducibility of the proposed high- and low-inflammatory endotypes described previously, which we characterize in this study. Further validation in larger training and validation cohorts is needed to confirm these findings. Moreover, future studies should utilize larger cohorts and aim to link molecular endotypes with treatment efficacy outcomes to evaluate their true clinical utility. Additionally, because this study did not assess longitudinally collected samples, it could only characterize an individual’s disease state at a single time point. Given that AD is characterized by periods of flares and remission, future studies tracking biomarker profiles over time may provide more insight into the potential dynamism of AD endotypes.

Overall, our study may contribute to understanding the heterogeneity among patients with AD. It makes preliminary and exploratory connections between the molecular mechanisms underlying AD endotypes and clinical measures such as EASI, eosinophil count, and cystatin C levels—parameters that, pending further functional validation, could be readily incorporated into clinical practice. Defining biomarker-based endotypes and their associations with clinical phenotypes represents an important starting point. Building on this research will help clinicians make more informed, personalized treatment decisions—particularly in the current landscape of increasingly targeted therapies for AD.

## Data Availability

Lilly provides access to all individual participant data collected during the trial, after anonymization, with the exception of pharmacokinetic or genetic data. Data are available to request 6 months after the indication studied has been approved in the US and EU and after primary publication acceptance, whichever is later. No expiration date of data requests is currently set once data are made available. In order to ensure appropriate use and analysis of data, access is provided after a proposal has been approved by an independent review committee identified for this purpose and after receipt of a signed data sharing agreement and will be provided as soon as reasonably possible. Data and documents, including the study protocol, statistical analysis plan, clinical study report, and blank or annotated case report forms, will be provided in a secure data sharing environment. For details on submitting a request, see the instructions provided at www.vivli.org. The raw numbers for charts and graphs are available in the source data file whenever possible. Source data are provided with this paper. All analyses in this publication were performed using readily available code in R. The packages and functions are referenced in the Methods and additional analysis details in the Supplementary material.
