# Supplementary material for: Comprehensive proteome profiling of molecular endotypes in Japanese adults with moderate-to-severe atopic dermatitis
Source: Front Med (Lausanne). 2025 Nov 20;12:1649918. doi: 10.3389/fmed.2025.1649918 (PMC12676969; doi:10.3389/fmed.2025.1649918)
Supplement: Supplementary file 1 [file Data_Sheet_1.pdf]

Supplementary Materials

Supplementary Methods

1. Inclusion criteria for the healthy control subjects

Healthy controls were confirmed to be non-allergic based on self-declaration during a screening process conducted by a healthcare professional at a clinical research site. The inclusion criteria were as follows:

a. No diagnosis of atopic dermatitis

b. No diagnosis of any allergic condition, inflammatory, or autoimmune disease

c. No self-declared allergies

d. No current diagnosis of cancer or chronic infections (including hepatitis B, hepatitis C, mycobacterial, or bacterial infections)

e. Not currently taking any medications

f. No fever (body temperature >37.5°C) or cold symptoms within 2 weeks prior to blood collection

g. No vaccinations within 2 weeks prior to blood collection

h. No self-reported seasonal allergies within 2 weeks prior to blood collection

2. Proteomic data QC criteria

All samples were run in a single batch, and each sample was assayed in singular. In the same batch, three internal controls (incubation, extension, and amplification) were included to monitor assay quality and performance, a plate control (pooled healthy serum) for data normalization, and a negative control to calculate the limit of detection for each assay and to assess the potential for contamination. Protein expression levels were given as normalized protein expression (NPX) values, which are arbitrary units used on a $\log_2$ scale that were normalized to the plate control. Post sequencing, the following criteria were applied as technical quality control (QC): 1) The average matched counts (the number of sequencing reads for each specific combination of sample and assay) for each sample had to be at least 500 counts, and 2) the deviation of the median value of the negative controls from a predefined value set for each assay had to be less than or equal to five standard deviations from the set predefined value. Assays not meeting the two criteria were flagged with a warning and were excluded from analysis.

For further data filtering, the following were applied: 1) only assays passing technical QC were included; 2) only analytes with NPX values above the limit of detection in more than 80% of samples were included; and 3) in cases of repeated protein analytes within the panel, one was randomly picked to avoid redundancy and potential data bias. To capture sufficient variation in protein expression, protein analytes were included only when their coefficient of variation (CoV) exceeded 20% (average CoV across all samples was 64.4%). After QC and filtering out proteins with low CoV, 1248 proteins were available for analysis.

3. WGCNA hyperparameters

Parameter	Input/Criteria
Network Type	Signed
Soft adjacency matrix threshold	9 (lowest power for which the scale-free topology model fit reached an R^2 of 0.9)
Module splitting (deepSplit)	4
Minimum module size	20
pamStage	TRUE (force all proteins into a module, i.e., no grey module)
Module merging threshold	0.25
Hub protein identification	Highest intramodular connectivity

4. Generation of modules for weighted gene co-expression network analysis. A scale-free, weighted, signed network was assumed, and a soft adjacency matrix was computed using a threshold of 9 (selected as the lowest power for which the scale-free topology model fit reached an R^2 of 0.9).

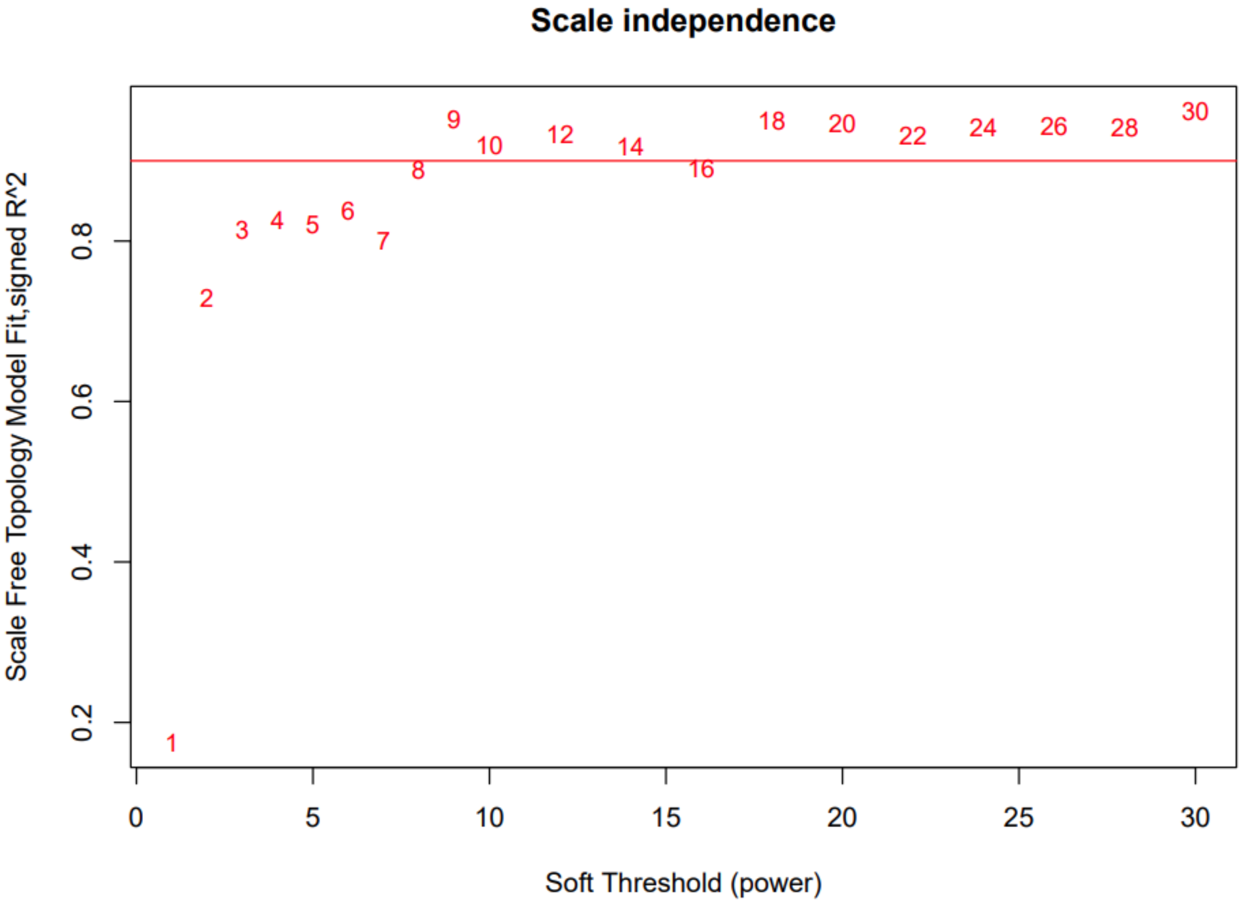

Table S1. AD clinical scores used for analysis.

**AD clinical scores**	**Definition**
AD.HI	*k*-means clustering outcome (0 = LO, 1 = HI)
ADSS	Atopic Dermatitis Symptom Score
Age	Patient age at time of trial entry
AGEDiag	Age at diagnosis
ALB	Serum albumin (g/dL)
ALP	Serum alkaline phosphatase (IU/L)
ALT	Serum alanine aminotransferase (IU/L)
AST	Serum aspartate aminotransferase (IU/L)
BMI	Body mass index
BSA	Body surface area
BUN	Serum blood urea nitrogen (mg/dL)
CHOL	Serum cholesterol (mg/dL)
CholtoHDL	Cholesterol-to-HDL ratio
Creat	Serum creatinine rate-blanked method (mg/dL)
CysC	Serum cystatin C immunoturbidimetric (mg/L)
DLQI	Dermatology Life Quality Index
DurDiag	Duration since first diagnosis

EASI	Eczema Area and Severity Index
eGFR	Estimated glomerular filtration rate (mL/min/1.73 m^2)
Eos	Blood eosinophils (× 10^9/L)
Flare1yr	Flare in the past year: yes = 1, no = 0
HADSAnx	Hospital Anxiety and Depression Scale – Anxiety
HADSDpr	Hospital Anxiety and Depression Scale – Depression
HDL	Serum HDL-cholesterol 3rd generation enzymatic method (mg/dL)
HGB	Blood hemoglobin (g/dL)
IGA	Investigator Global Assessment
IgA	Serum immunoglobulin A nephelometry (mg/dL)
IgE	Serum immunoglobulin E (IU/mL)
IgG	Serum immunoglobulin G nephelometry (mg/dL)
IgM	Serum immunoglobulin M nephelometry (mg/dL)
ItchNRS	Itch Numeric Rating Scale
LDL	Serum LDL-cholesterol Friedewald calculation (mg/dL)
LYM	Blood lymphocytes (× 10^9/L)
NEUT	Blood neutrophils (× 10^9/L)
PainNRS	Pain Numeric Rating Scale

PGI.S.AD	Patient Global Impression of Severity
Platelet	Blood platelets (× 10^9/L)
POEM	Patient-Oriented Eczema Measure
PreBio	Prior biologics
PreCyc	Prior cyclosporin
PreSys	Prior systemic therapy
PreSysCS	Prior systemic corticosteroid
PreSysImSu	Prior systemic immune suppressant
PreTCS	Prior topical corticosteroid
PreTCNI	Prior topical calcineurin inhibitor
PROT	Serum protein (g/dL)
SCORAD	SCORing Atopic Dermatitis score
SEX	Sex
TRIG	Serum triglycerides (mg/dL)
Urate	Serum urate (mg/dL)
WBC	Blood leukocytes (× 10^9/L)
Weight	Weight (kg)

Abbreviations: AD, atopic dermatitis; calc., calculation; HDL, high-density lipoprotein; HI, high; LDL, low-density lipoprotein; LO, low; WBC, white blood cell.

Table S2. Differentially expressed proteins in patients with AD compared to HCs.

Protein	Fold change* (logFC)	Outcome
EGF	4.65	Upregulated_AD
EREG	3.76	Upregulated_AD
CCL17	3.70	Stepwise
SKAP2	3.69	Upregulated_AD
CD40LG	3.56	Upregulated_AD
CRACR2A	3.14	Upregulated_AD
SNAP23	3.13	Upregulated_AD
MPIG6B	3.12	Upregulated_AD
CORO1A	3.03	Stepwise
FKBP5	2.85	Upregulated_AD
CD69	2.84	Upregulated_AD
SRC	2.76	Upregulated_AD
PDLIM7	2.73	Upregulated_AD
RNASE3	2.62	Stepwise
IL-19	2.62	Stepwise
CA13	2.61	Upregulated_AD
DBNL	2.60	Upregulated_AD

Protein	Fold change* (logFC)	Outcome
DEFB4A_DEFB4B	2.58	Upregulated_AD
PMVK	2.54	Upregulated_AD
DOK2	2.50	Upregulated_AD
DIABLO	2.49	Upregulated_AD
TNFSF14	2.48	Upregulated_AD
MAP2K6	2.47	Upregulated_AD
DNAJA2	2.41	Upregulated_AD
PTPN1	2.40	Upregulated_AD
GP6	2.39	Upregulated_AD
CRKL	2.35	Upregulated_AD
SNAP29	2.27	Upregulated_AD
CCL26	2.26	Stepwise
LGALS7_LGALS7B	2.21	Stepwise
TBCB	2.21	Stepwise
KRT5	2.21	Stepwise
ARHGEF12	2.19	Upregulated_AD

Protein	Fold change* (logFC)	Outcome
GYS1	2.18	Upregulated_AD
CALCOCO1	2.15	Upregulated_AD
EDAR	2.13	Upregulated_AD
CCL18	2.12	Stepwise
FYB1	2.10	Upregulated_AD
BIN2	2.06	Upregulated_AD
CCL13	2.05	Upregulated_AD
ARSB	2.05	Upregulated_AD
SLC27A4	2.05	Upregulated_AD
TYMP	2.04	Stepwise
CCL22	2.04	Stepwise
TBC1D23	2.04	Upregulated_AD
MMP8	2.03	Stepwise
YES1	2.03	Upregulated_AD
OSM	2.01	Upregulated_AD
SULT1A1	1.99	Upregulated_AD
CCL7	1.98	Stepwise

Protein	Fold change* (logFC)	Outcome
WFDC12	1.98	Upregulated_AD
CASP8	1.96	Upregulated_AD
DPP7	1.91	Upregulated_AD
CC2D1A	1.90	Upregulated_AD
CASP2	1.90	Upregulated_AD
SCAMP3	1.89	Upregulated_AD
EIF4B	1.84	Upregulated_AD
OLR1	1.84	Stepwise
LBR	1.84	Stepwise
MGLL	1.83	Upregulated_AD
PRDX5	1.82	Upregulated_AD
SUSD1	1.81	Upregulated_AD
CIAPIN1	1.80	Upregulated_AD
GLB1	1.80	Upregulated_AD
STX4	1.80	Upregulated_AD
CCL27	1.79	Stepwise
TGFA	1.79	Upregulated_AD

Protein	Fold change* (logFC)	Outcome
PVALB	1.79	Upregulated_AD
PLXNA4	1.79	Upregulated_AD
IRAG2	1.78	Stepwise
ERBIN	1.77	Upregulated_AD
AZU1	1.77	Stepwise
CLEC1B	1.74	Upregulated_AD
MMP9	1.73	Upregulated_AD
PADI4	1.73	Upregulated_AD
BTC	1.72	Upregulated_AD
EIF4G1	1.72	Stepwise
MAP3K5	1.71	Upregulated_AD
ATOX1	1.68	Upregulated_AD
CLC	1.68	Stepwise
MNDA	1.68	Stepwise
TNC	1.66	Stepwise
PSIP1	1.65	Upregulated_AD
IL-13	1.65	Stepwise

**Protein**	**Fold change* (logFC)**	**Outcome**
TBC1D5	1.65	Upregulated_AD
TMSB10	1.65	Stepwise
TACC3	1.63	Upregulated_AD
FADD	1.62	Upregulated_AD
MANF	1.61	Upregulated_AD
IL-32	1.61	Upregulated_AD
CDKN2D	1.60	Upregulated_AD
SDC4	1.60	Upregulated_AD
CASP10	1.60	Upregulated_AD
ATP5IF1	1.58	Upregulated_AD
MPO	1.58	Stepwise
BANK1	1.57	Upregulated_AD
MMP12	1.57	Stepwise
DPY30	1.56	Stepwise
DNMBP	1.55	Upregulated_AD
ANXA11	1.54	Upregulated_AD
DCTPP1	1.53	Stepwise

Protein	Fold change* (logFC)	Outcome
CDKN1A	1.53	Upregulated_AD
BAX	1.53	Upregulated_AD
F11R	1.51	Upregulated_AD
CKMT1A_CKMT1B	1.51	Upregulated_AD
IRAK1	1.51	Upregulated_AD
SAMD9L	1.51	Upregulated_AD
CASP3	1.51	Upregulated_AD
TRIM21	1.48	Upregulated_AD
NT5C3A	1.48	Upregulated_AD
ILKAP	1.48	Upregulated_AD
CEACAM8	1.47	Upregulated_AD
ZBTB17	1.46	Stepwise
S100A12	1.46	Stepwise
PI3	1.46	Stepwise
LAT2	1.43	Upregulated_AD
DAG1	1.43	Upregulated_AD
NID2	1.43	Upregulated_AD

**Protein**	**Fold change* (logFC)**	**Outcome**
FEN1	1.42	Stepwise
CLIP2	1.40	Upregulated_AD
NSFL1C	1.40	Upregulated_AD
DSG3	1.38	Upregulated_AD
PAG1	1.38	Upregulated_AD
NFATC1	1.37	Upregulated_AD
SEMA4D	1.36	Upregulated_AD
INPP1	1.36	Upregulated_AD
BAG3	1.36	Upregulated_AD
APBB1IP	1.35	Stepwise
CLEC4D	1.35	Upregulated_AD
PRTN3	1.34	Stepwise
SIAE	1.34	Upregulated_AD
SERPINB8	1.34	Upregulated_AD
IL-16	1.34	Upregulated_AD
METAP2	1.34	Upregulated_AD
LY6D	1.34	Upregulated_AD

Protein	Fold change* (logFC)	Outcome
RNF41	1.33	Upregulated_AD
CRADD	1.33	Upregulated_AD
MYO9B	1.33	Upregulated_AD
HSPA1A	1.32	Upregulated_AD
DFFA	1.32	Upregulated_AD
NADK	1.31	Stepwise
PLIN3	1.31	Upregulated_AD
CASP1	1.31	Stepwise
TJAP1	1.31	Upregulated_AD
IL-6	1.30	Upregulated_AD
TARBP2	1.30	Upregulated_AD
IQGAP2	1.29	Upregulated_AD
PPP1R2	1.29	Stepwise
VSIR	1.28	Upregulated_AD
DARS1	1.27	Upregulated_AD
AXIN1	1.27	Upregulated_AD
BLMH	1.27	Upregulated_AD

Protein	Fold change* (logFC)	Outcome
ENO1	1.26	Upregulated_AD
STAT5B	1.26	Stepwise
SETMAR	1.26	Upregulated_AD
ICA1	1.26	Upregulated_AD
TNFRSF8	1.26	Upregulated_AD
ANGPT1	1.25	Upregulated_AD
HBEGF	1.25	Upregulated_AD
STX16	1.25	Upregulated_AD
FKBP4	1.24	Upregulated_AD
MAEA	1.24	Upregulated_AD
SIRT2	1.23	Upregulated_AD
PPIB	1.23	Upregulated_AD
PRKAB1	1.23	Upregulated_AD
MAD1L1	1.22	Stepwise
SERPINB1	1.22	Stepwise
HSPB1	1.21	Upregulated_AD
THOP1	1.21	Upregulated_AD

Differential protein expression between patients with AD and HCs was assessed using a linear model with age and sex as covariates (R package: limma) (17). Adjusted p-values were calculated using the Benjamini-Hochberg method (18). *All fold-change values shown are $p<0.05$ for patients with AD versus HCs.

Abbreviations: AD, atopic dermatitis; HC, healthy control.

Table S3. Differentially expressed proteins in the AD_HI versus AD_LO endotype.

Protein	Fold change* (logFC)	Outcome
IL-19	1.82	Stepwise
STAT5B	1.70	Stepwise
CCL17	1.67	Stepwise
NCF2	1.66	AD_HI_Unique
TNC	1.64	Stepwise
RASSF2	1.62	AD_HI_Unique
PI3	1.62	Stepwise
MNDA	1.60	Stepwise
CLC	1.59	Stepwise
S100A12	1.55	Stepwise
FEN1	1.54	Stepwise
CCL22	1.51	Stepwise
TMSB10	1.51	Stepwise
HNRNPK	1.51	AD_HI_Unique
CCL26	1.48	Stepwise
CCL18	1.48	Stepwise
RNASE3	1.47	Stepwise

Protein	Fold change* (logFC)	Outcome
CORO1A	1.47	Stepwise
TYMP	1.44	Stepwise
APEX1	1.42	AD_HI_Unique
ZBTB17	1.38	Stepwise
EGLN1	1.37	AD_HI_Unique
NBN	1.36	AD_HI_Unique
HDGF	1.36	AD_HI_Unique
MMP12	1.36	Stepwise
APBB1IP	1.35	Stepwise
MMP8	1.35	Stepwise
PXN	1.35	AD_HI_Unique
CCL7	1.34	Stepwise
KRT5	1.33	Stepwise
DPY30	1.33	Stepwise
LBR	1.33	Stepwise
TBCB	1.33	Stepwise
IL-13	1.32	Stepwise

**Protein**	**Fold change* (logFC)**	**Outcome**
LGALS7_LGALS7B	1.32	Stepwise
PFKFB2	1.31	AD_HI_Unique
PRTN3	1.30	Stepwise
MGMT	1.30	AD_HI_Unique
NADK	1.30	Stepwise
AZU1	1.30	Stepwise
NMNAT1	1.29	AD_HI_Unique
FMNL1	1.28	AD_HI_Unique
PTPN6	1.27	AD_HI_Unique
CCL27	1.27	Stepwise
CDH3	1.26	AD_HI_Unique
OLR1	1.25	Stepwise
DCTPP1	1.24	Stepwise
SERPINB1	1.24	Stepwise
MAD1L1	1.24	Stepwise
SRP14	1.23	AD_HI_Unique
IRAG2	1.23	Stepwise

Protein	Fold change* (logFC)	Outcome
CASP1	1.22	Stepwise
MPO	1.21	Stepwise
EIF4G1	1.21	Stepwise
PPP1R2	1.21	Stepwise
NDRG1	1.21	AD_HI_Unique

Differential protein expression between the AD_HI and AD_LO clusters was assessed using a linear model with age and sex as covariates (R package: limma) (17). Adjusted p-values were calculated using the Benjamini-Hochberg method (18). *All fold-change values shown are $p<0.05$ for AD_HI versus AD_LO.

Abbreviations: AD, atopic dermatitis; HI, high; LO, low.

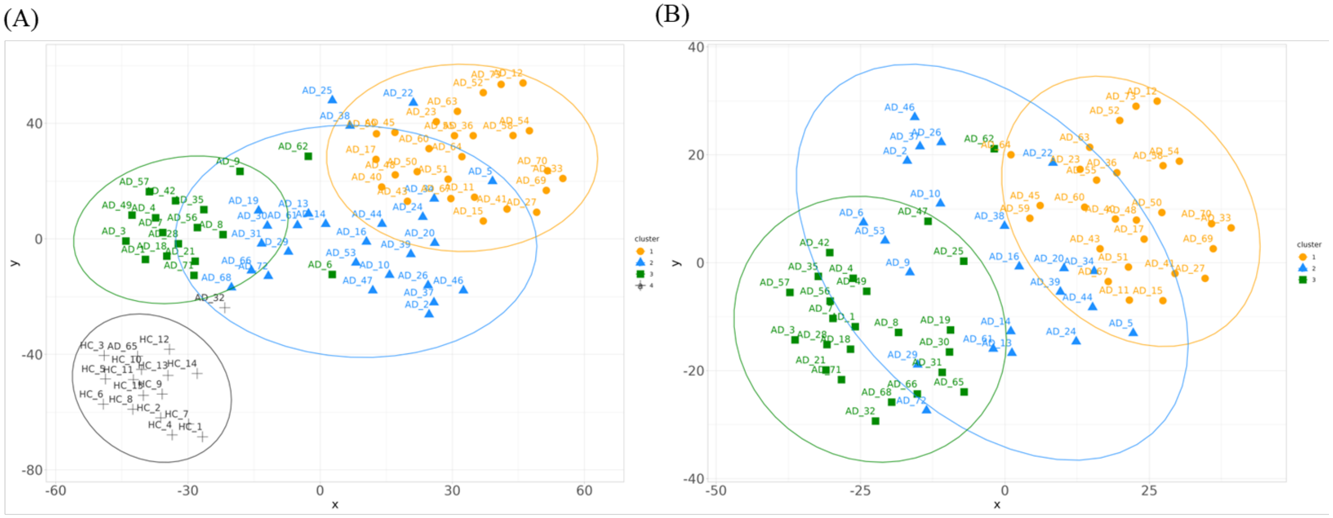

Confusion matrix		k=4, all data			
		Cluster 1	Cluster 2	Cluster 3	Cluster 4
k=3, patient data	Cluster 1	27	0	0	0
	Cluster 2	0	20	2	0
	Cluster 3	0	7	15	2

Figure S1. *t*-SNE projections of clusters derived from (A) K4_alldata and (B) K3_patientdata.

Cluster 1 appeared stable, but clusters 2 and 3 contained different assignments when HCs were excluded from the analysis.

Abbreviations: AD, atopic dermatitis; HC, healthy control; *t*-SNE, *t*-distributed stochastic neighbor embedding

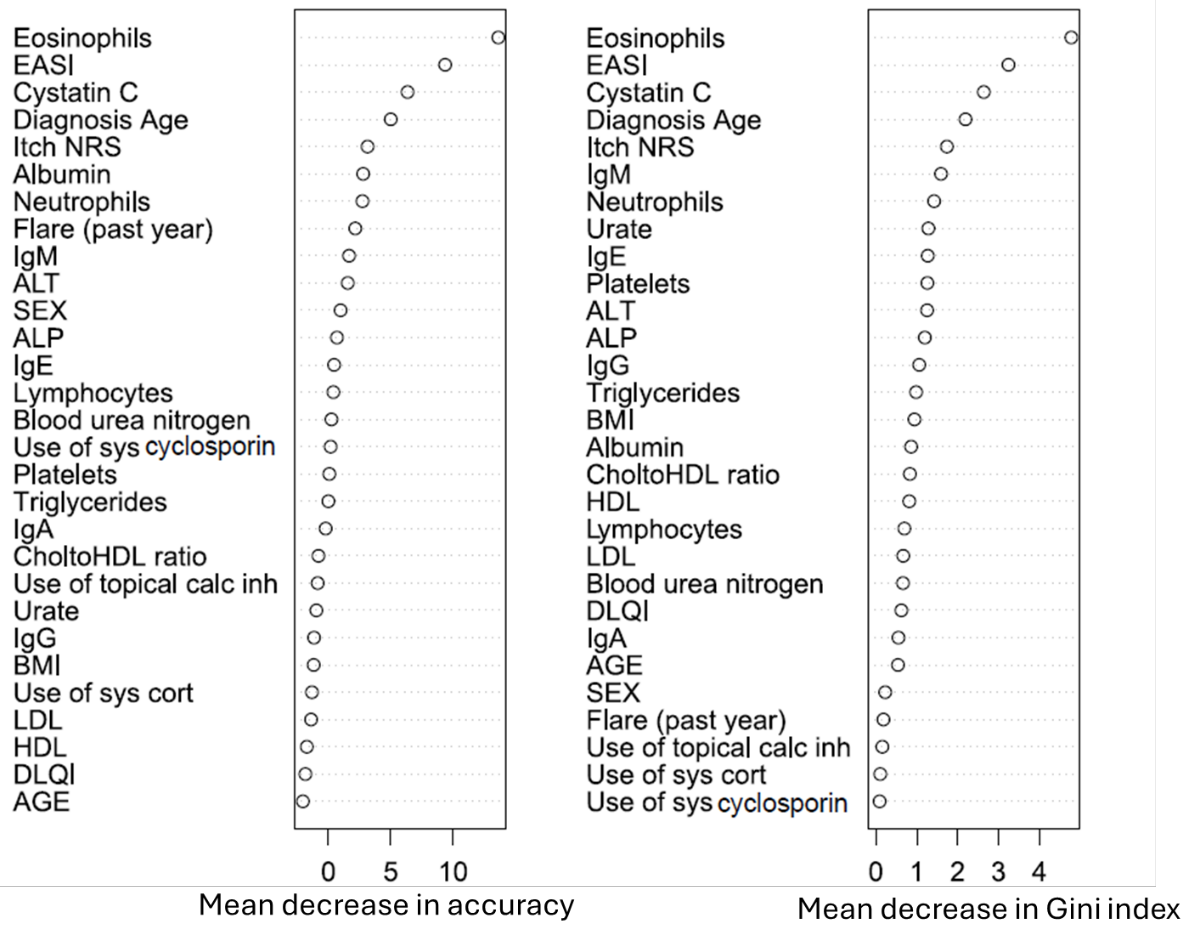

Figure S2. Top predictors of clusters from a clinical data model generated by random forest analysis.

Abbreviations: ALP, alkaline phosphatase; ALT, alanine aminotransferase; BMI, body mass index; calc, calcineurin; chol, cholesterol; cort, corticosteroid; DLQI, Dermatology Life Quality Index; EASI, Eczema Area and Severity Index; HDL, high-density lipoprotein; Ig, immunoglobulin; inh, inhibitor; LDL, low-density lipoprotein; NRS, Numeric Rating Scale; sys, systemic.

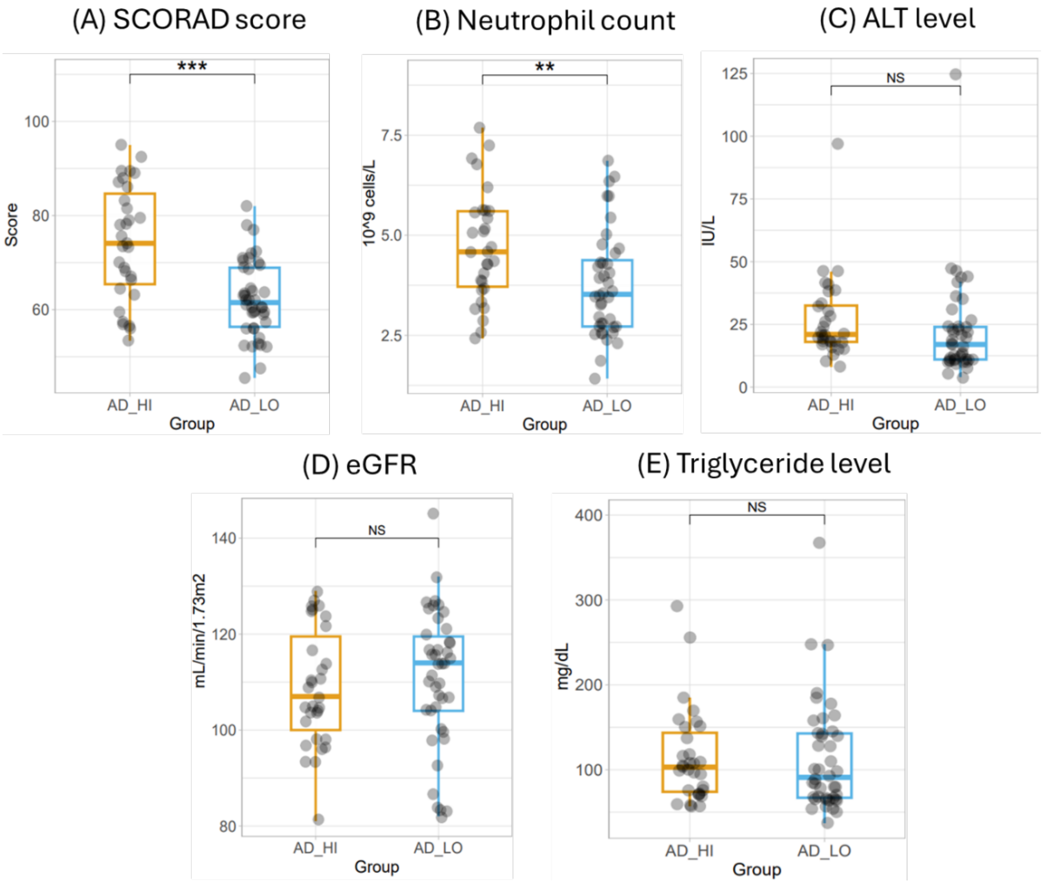

Figure S3. Box plots for the AD_HI and AD_LO endotypes for (A) SCORAD score, (B) neutrophil count, (C) ALT levels, (D) eGFR, and (E) triglyceride levels.

Inflammatory biomarkers were upregulated in the AD_HI endotype, which is associated with disease severity. P-values were based on the Wilcoxon rank-sum test. *p<0.05, **p<0.01, ***p<0.001. Abbreviations: AD, atopic dermatitis; ALT, alanine aminotransferase; eGFR, estimated glomerular filtration rate; HI, high;; LO, low; NS, not significant; SCORAD, SCORing Atopic Dermatitis

Ethics Review Board Information for the BREEZE AD2 trial (study sites in Japan)

Dr Mano Medical Clinic, 1-8-1 Ebisu, Shibuya-ku, Tokyo, 150-0013, Japan

Gifu University Hospital, 1-1 Yanagido, Gifu, Gifu, 501-1194, Japan

Hayashi Diabetes Internal Medicine, Abeasa Medical 2f, 3-2, Shinei-cho, Chigasaki-shi, Kanagawa, 253-0044, Japan

Ihl Shinagawa East One Medical Clinic Institutional Review Board, 2-16-1, Kounan, East One Tower 3f, Minato-ku, Tokyo, 108-0075, Japan

Jichi Medical University Hospital, 3311-1 Yakushiji, Shimotsuke, Tochigi, 329-0498, Japan

Kurume University Hospital, 67 Asahi-machi, Kurume, Fukuoka, 830-0011, Japan

Osaka City University Hospital, 1-5-7 Asahimachi, Abeno-ku, Osaka, Osaka, 545-8586, Japan

Osaka Habikino Medical Center, 3-7-1 Habikino, Habikino, Osaka, 583-8588, Japan

Sapporo Dermatology Clinic Institutional Review Board, 2-1-1, Minami3-jonishi, Chuo-ku Sapporo-shi, Hokkaido, 060-0063, Japan

Sapporo Skin Clinic, 2-1-1 Nishi, Minami 3jo, Chuo-ku, Sapporo, Hokkaido, 060-0063, Japan

Tokyo Medical University Ibaraki Medical Center, 3- 20-1 Amimachi Chuo, Inashiki-gun, Ibaraki, 300-0395, Japan

Tokyo Teishin Hospital, 2-14-23, Fujimi, Chiyoda-ku, Tokyo, 102-8798, Japan

Ethics Review Board Information for the Healthy Control samples

OPHAC Hospital of Heishinkai Medical Corporation Investigational Review Board

4-1-29 Miyahara, Yodogawa-ku, Osaka-shi, Osaka, 583-8588, Japan
